# Editorial: Bone integrity in patients with osteoporosis: Evaluation of fracture risk and influence of pharmacological treatments and mechanical aspects

**DOI:** 10.3389/fbioe.2023.1232257

**Published:** 2023-06-08

**Authors:** Javier Martínez-Reina, Peter Pivonka, Ridha Hambli

**Affiliations:** ^1^ Departamento de Ingeniería Mecánica y Fabricación, Universidad de Sevilla, Seville, Spain; ^2^ School of Mechanical, Medical and Process Engineering, Queensland University of Technology, Brisbane, QLD, Australia; ^3^ Polytech Orléans, University of Orleans, Orleans, France

**Keywords:** osteoporosis diagnostic tool, meta-analysis, bone cell population models, 3D shaper, asiatic acid, denosumab, bisphosphonate, PK-PD model

Osteoporosis (OP) is one of the most common metabolic bone diseases and OP related fragility fractures represent a costly socio-economic burden worldwide, for its high incidence and the severe consequences to elderly people. Treatments aim at reducing the fracture risk by trying to recover the mechanical integrity of bone to pre-disease state. This is done by reversing the effect of the metabolic disorders caused by factors such as ageing, oestrogen deficiency, prolonged exposure to glucocorticoids or hyperparathyroidism on bone remodelling.

Drug treatments include anti-catabolic therapies, anabolic therapies or both. Anabolic drugs are preferred over anti-catabolic drugs as the latter tend to result in excessively mineralised and brittle tissue, which usually leads to low-energy fractures in the long term. For this reason, anti-catabolic treatments are interrupted after a certain time, though it has been reported that discontinuation produces a rebound of bone loss and associated fractures, whose causes still remain unclear. These facts together pose a dilemma that is still unresolved and is being addressed from different points of view: multiple drug therapies (sequential vs. combination), patient-specific treatments, or addition of exercise to the therapy, among others.

Osteoporotic fractures are usually low-trauma which makes them occur inadvertently and emphasizes the importance of early diagnosis. Much effort has been focused on the development of techniques to assess the fracture risk, from the simplistic and already outdated FRAX, to more recent and powerful computational tools, based on image analysis and bone histomorphometry.

The works presented in this Research Topic are varied and interesting, covering aspects ranging from basic research to the use of artificial intelligence, image processing and *in silico* tests. They constitute a good sample of the research being carried out nowadays, both in the diagnosis and treatment of osteoporosis, and may contribute to open new lines of future research.

A cutting-edge diagnostic technique involves the application of artificial intelligence to radiographic texture analysis. Cui et al. proposed in this issue a support vector machine classifier synthesizing pixel-wise fractal, entropy, and global lacunarity. The new tool performed better than previously proposed classifiers and was able to distinguish between three levels of bone loss in lumbar spine: normal, osteopenia, and osteoporosis. In addition, it showed high generalization on osteoporosis diagnosis using lateral radiographs of the calcaneus. This tool could help clinicians evaluate radiographic studies to avoid overlooking of asymptomatic osteoporosis due to human errors.

The above paper discusses the most common method today for diagnosing osteoporosis, areal bone mineral density (aBMD) computed from 2D DXA scans. However, aBMD is a limited surrogate for femoral strength since it does not account for 3D bone geometry and density distribution. QCT scans allow such 3D reconstruction to build a finite element (FE) model that can deliver improved femoral strength predictions. However, QCT implies a higher radiation dose and higher costs that prevent a systematic usage of this technique for screening purposes. The recently launched 3D-Shaper ®software reconstructs the 3D shape and density distribution from 2D DXA scans and could be a valuable tool if proven reliable. In this Research Topic Dudle et al. presented the first independent evaluation of the software. The authors showed that 3D-Shaper generates an altered BMD distribution compared to QCT but shows an interesting potential for deriving a standardized femoral strength from a 2D DXA scan.

Regarding OP treatments, research continues on new drugs or on the effects of already existing drugs. This Research Topic includes works of both types. Dong et al. analysed the anti-catabolic effect of asiatic acid (AA), a natural compound extracted from a traditional Chinese herb. AA inhibits NF-kappaB/MAPK/Protein kinase B signaling pathway, as well as the downstream factors of NFATc1 in the osteoclast signalling pathway activated by RANKL. The *in vivo* experiments suggested that AA could alleviate ovariectomy-induced bone loss in mice; however, it did not significantly promote osteoblast differentiation and mineralisation.

On the other hand, Wang et al. reviewed the recent advances in the research on zoledronic acid (ZA). This widely prescribed bisphosphonate inhibits osteoclastogenesis and bone resorption by suppressing the canonical RANKL/RANK signalling pathway and the non-canonical Wnt pathway. Additionally, ZA induce osteoclast apoptosis by inhibiting the effect of farnesyl pyrophosphate synthase (FPPS), increasing the concentrations of reactive oxygen species (ROS), and through ferroptosis, a newly discovered iron-mediated cell death.

Basic research on the mechanisms of action of OP drugs is key in their development but their efficacy must ultimately be assessed in clinical studies. Many of them can be found in the literature on each drug, carried out by different groups. In this case, a meta-analysis can help increase the accuracy of the results, by increasing the size of the study. In this Research Topic two meta-analyses are presented: one on bisphosphonates (Li et al.), addressing different drugs of this group, and one on the sequential combination of an anabolic (teriparatide) and an anti-catabolic drug (denosumab) (Sun et al.).

Sequential combination of drugs is receiving much attention from the scientific community since it may constitute a solution to one important issue of treatments: the impossibility of extending single-drug treatments because of long-term adverse effects. For certain drugs a significant rebound in bone loss is observed upon discontinuation. In the case of denosumab, this discontinuation is analysed by Martínez-Reina et al., who also proposed some strategies to follow after discontinuation, including the sequential combination with bisphosphonates. They performed *in silico* simulations of the treatments using a bone cell populations model (BCPM). These type of simulations have several advantages over clinical studies: the time needed to conduct the study, the combinations of parameters that can be analysed and most importantly, the absence of ethical implications.

Another *in silico* study is presented by Calvo-Gallego et al. who addressed OP treatment with alendronate. These authors developed a pharmacokinetics-pharmacodynamics model of alendronate coupled to a BCPM in order to predict the bone gain achieved with two dosing regimens: 70 mg weekly and 10 mg daily. The novelty of this model lies in how it takes into account drug accumulation in the bone matrix and drug release by osteoclasts during resorption.

As has been shown in this Research Topic, great advances have been made in better understanding the aetiology, progression and treatment of osteoporosis both in terms better imaging modalities, bone biomarkers and more recently *in silico* simulations of osteoporosis treatments. We believe that particularly *in silico* simulations have the potential to provide further insights into efficacy of drug treatments which are becoming increasingly complex in terms of use of multiple drugs concurrently or sequentially. Using such tools will help clinicians to design patient specific treatments that will allow to optimise dose and timing of drug administration.

